# Contribution of Disruption in Creatine Synthesis and Transporter to 6-PPD Quinone Induced Immunosuppression in *Caenorhabditis elegans*

**DOI:** 10.3390/toxics14070601

**Published:** 2026-07-09

**Authors:** Dayu Hu, Bingying Li, Dayong Wang

**Affiliations:** Jiangsu Provincial Key Laboratory of Critical Care Medicine, Medical School, Southeast University, Nanjing 210009, China

**Keywords:** 6-PPDQ, creatine synthesis, transporter, ATP, immunosuppression, *C. elegans*

## Abstract

6-PPD quinone (6-PPDQ) has been recognized as a typical emergent contaminant with the potential to cause multiple aspects of damage to organisms. Creatine is an important metabolite that mediates energy homeostasis. In *Caenorhabditis elegans*, creatine content was reduced by 0.1–10 μg/L of 6-PPDQ, which was accompanied by a decrease in the expression of *argk-1* encoding a creatine kinase and *snf-5* encoding a potential creatine transporter. Creatine content could be reduced by RNAi of *argk-1* and *snf-5*. Moreover, RNAi of *argk-1* and *snf-5* aggravated 6-PPDQ-induced immunosuppression, reflected by decreased expression of antimicrobial genes (*lys-7* and *spp-1*), and double RNAi of *argk-1* and *snf-5* resulted in more severe immunosuppression induction in 6-PPDQ-exposed nematodes. After 6-PPDQ exposure, RNAi of *argk-1* and *snf-5* decreased the expression of *aak-2* encoding AMPK, and *aak-2* RNAi also strengthened 6-PPDQ-induced immunosuppression. During control of 6-PPDQ caused immunosuppression, ARGK-1, SNF-5, and AAK-2 modulated expressions of PMK-1/p38 MAPK and DAF-16 signals. The immunosuppression and inhibition in PMK-1 and DAF-16 expressions induced by 6-PPDQ could be further suppressed by creatine treatment. Therefore, the environmental exposure risk of 6-PPDQ in disrupting creatine synthesis and transporter was suggested, which potentially contributes to the induction of immunosuppression in nematodes.

## 1. Introduction

N-(1,3-Dimethylbutyl)-N’-phenyl-p-phenylenediamine (6-PPD) is a widely used p-phenylenediamine antioxidant in tire rubber with the aim of inhibiting rubber cracking and aging and extending service life [1]. During tire wear and aging, 6-PPD can be released into environments and further transformed into 6-PPD quinone (6-PPDQ) under the action of ozone and other oxidants [2,3]. This transformation product was initially identified as a key pollutant responsible for acute mortality of sensitive salmonid species [4]. After that, 6-PPDQ was detected in different environmental matrices, including urban and road runoff, rivers and streams, sediments, and airborne particulate matter, suggesting its broad environmental occurrence [5,6]. In surface water and runoff, environmental concentrations of 6-PPDQ are generally detected at levels ranging from ng/L to tens of μg/L [7,8]. Detection of 6-PPDQ in human blood, urine, and cerebrospinal fluid indicates potential internal exposure of humans to this emerging contaminant and health risks [9,10]. Exposure to 6-PPDQ can cause multiple toxic effects on organisms, including disruption in metabolism [11,12,13].

*Caenorhabditis elegans* is a useful animal model for evaluating pollutant-induced toxicity due to its high sensitivity to environmental exposures [14,15]. With the aid of well-described molecular backgrounds, this animal model can be used to elucidate toxicological mechanisms of observed toxic effects after pollutant exposure [16,17]. In *C. elegans*, initially, some aspects of toxicity on organs were observed after 6-PPDQ exposure, such as damage to the intestine and nervous system [18,19,20]. Exposure to 6-PPDQ also resulted in lifespan reduction [21,22] and immunosuppression reflected by a decrease in expression of antimicrobial genes (*lys-7* and *spp-1*) [23,24]. Moreover, some metabolic processes, such as amino acid metabolism, can be disrupted by 6-PPDQ [25,26,27,28]. These observations imply the possible link between metabolic disruption and 6-PPDQ-induced toxicity, such as immunosuppression.

**Figure 1:**
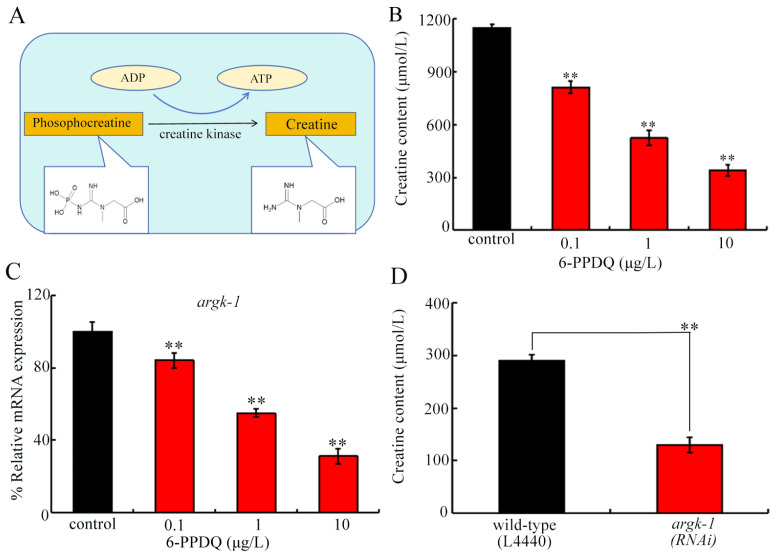
Effect of 6-PPDQ exposure on creatine content. (**A**) A diagram showing creatine synthesis. (**B**) Effect of 6-PPDQ exposure on creatine content. Control, without 6-PPDQ exposure. ** *p* < 0.01 vs. control. (**C**) Effect of 6-PPDQ exposure on *argk-1* expression. ** *p* < 0.01 vs. control. Control, without 6-PPDQ exposure. (**D**) Effect of RNAi of *argk-1* on creatine content in 6-PPDQ-exposed nematodes. Exposure concentration of 6-PPDQ was 10 μg/L. ** *p* < 0.01.

Creatine is an important metabolite generated from phosphocreatine to mediate the energy homeostasis [29]. The creatine participated in the regulation of immunity [30,31]. In *C. elegans*, creatine kinase ARGK-1 participated in high-energy phosphate transfer (Figure 1A), which was associated with AAK-2/AMPK activation [32]. SLC6A8 is a major creatine transporter in mammals, and the *C. elegans* sodium-dependent neurotransmitter/solute transporter family (SNF) is the corresponding homolog [33,34]. For example, SNF-5 was annotated as a homolog of SLC6A8 to be involved in transmembrane transport of metabolites, including creatine [35,36]. We assumed that disruption in creatine synthesis may contribute to the formation of 6-PPDQ-induced immunosuppression. Using *C. elegans* as an animal model, we thus first investigated the effect of 6-PPDQ exposure on creatine synthesis and transport. Moreover, the roles of ARGK-1 and creatine transporter in regulating 6-PPDQ-induced immunosuppression and the underlying mechanism were examined. Our findings provide an important basis for the association of disruption in creatine metabolism with 6-PPDQ-induced immunosuppression.

## 2. Materials and Methods

### 2.1. Animal Maintenance

The wild-type N2 strain was used as the primary strain for toxicity assessment in this study, and genotype information of *C. elegans* strains used in the experiments is listed in Table S1. For routine maintenance, all nematode strains were cultured on nematode growth medium (NGM) plates seeded with *E. coli* OP50 and maintained at 20 °C under standard conditions [37].

To ensure developmental synchronization, nematodes were synchronized using a standard embryo isolation procedure [38]. The isolated embryos were then hatched to obtain synchronized L1-arrested larvae, which were subsequently used for the exposure experiments.

### 2.2. Exposure

Concentrations of 0.1–10 μg/L were selected for 6-PPDQ, which reflect environmentally relevant concentrations (ERCs) in aquatic systems [4,8]. Synchronized L1 larvae were exposed to 6-PPDQ solutions for 6.5 days until they reached the third day of adulthood [39]. To reduce fluctuations in chemical concentrations and ensure sufficient nutritional supply during exposure, 6-PPDQ solutions were freshly made daily, and OP50 was added to the solutions.

### 2.3. Creatine Content

Creatine levels were measured using a commercial assay kit (Shanghai Beyotime Biotechnology Co., Shanghai, China). Nematodes were collected, weighed, and homogenized. After centrifugation, supernatants were collected for subsequent analysis. For each reaction, 20 μL of sample supernatant or creatine standard was added to a 96-well plate, followed by 80 μL of freshly prepared Amplex Red working solution containing creatine assay buffer, Amplex Red, enzyme solution A, and enzyme solution B. Absorbance of reaction mixtures was measured at 570 nm. Creatine concentrations were calculated based on a standard curve generated with kit-provided standards. Experiments were repeated three times.

### 2.4. Lifespan

Lifespan is an indirect indicator of innate immune response in *C. elegans* [40]. Exposure to 6-PPDQ could decrease expression of antimicrobial genes (*spp-1* and *lys-7*) [23], and RNAi of *spp-1* and *lys-7* strengthened lifespan reduction induced by 6-PPDQ [24]. Lifespan reduction can be used to reflect the toxic effects caused by immunosuppression induced by 6-PPDQ in nematodes. In the lifespan assay, 50 nematodes were randomly placed onto a plate for each treatment and scored every day. To prevent interference from spawning, nematodes were transferred daily. Dead nematodes were identified by their lack of response to platinum wire stimuli. Median lifespan indicates the time when the survival rate is 50% [41]. Experiments were repeated three times. Survival data were statistically analyzed using the Kaplan–Meier method (SPSS software v.27).

### 2.5. Gene Expression

TRIzol reagent was used for total RNA isolation. Concentration and purity of RNA were determined by measuring absorbance at 260 and 280 nm. After chloroform extraction and phase separation, the aqueous supernatant containing RNA was transferred to a fresh tube. cDNA was synthesized using M-MulV reverse transcriptase. Gene expression levels were subsequently quantified by qRT-PCR with *tba-1* as a reference gene [42]. Experiments were repeated three times. All primer sequences are listed in Table S2.

### 2.6. RNA Interference (RNAi)

Gene knockdown was performed using the RNAi feeding method [43]. RNAi plasmids containing target gene fragments or empty vector L4440 were transformed into *E. coli* HT115. After IPTG induction, corresponding bacterial cultures were seeded onto NGM plates for nematode feeding. Synchronized L1 larvae were transferred onto the RNAi bacterial lawns and cultured. The progeny was then collected and used for subsequent 6-PPDQ exposure experiments. Knockdown efficiency was confirmed by qRT-PCR (Figure S1).

### 2.7. Pharmacological Treatment

Following 6-PPDQ exposure (10 μg/L), nematodes were transferred into and treated in 5 mM creatine solution from day 3 of adulthood for 24 h [44]. Experiments were conducted in triplicate.

### 2.8. Data Analysis

Data were presented as mean ± standard deviation (SD). Statistical analysis was conducted using GraphPad Prism (v8). For comparisons involving more conditions, ANOVA was performed, followed by Tukey’s test. When comparing two conditions, an unpaired Student’s *t*-test was utilized. A *p*-value of <0.01 (**) indicates statistical significance.

## 3. Results

### 3.1. 6-PPDQ Inhibited the Creatine Synthesis

In *C. elegans*, the ortholog of mammalian creatine kinase ARGK-1 catalyzes creatine synthesis from phosphocreatine (Figure 1A) [32]. Creatine content was reduced by 0.1–10 μg/L 6-PPDQ (Figure 1B). Meanwhile, *argk-1* expression was decreased by 0.1–10 μg/L 6-PPDQ (Figure 1C). After 6-PPDQ exposure, RNAi of *argk-1* could reduce creatine content (Figure 1D).

### 3.2. 6-PPDQ Inhibited Potential Creatine Transporter

**Figure 2:**
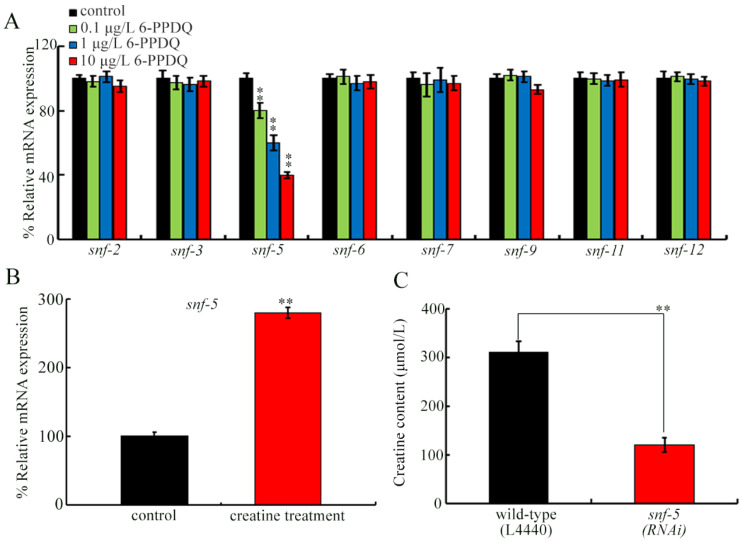
Effect of 6-PPDQ exposure on potential creatine transporters. (**A**) Effect of 6-PPDQ exposure on expression of potential creatine transporter genes. Control, without 6-PPDQ exposure. ** *p* < 0.01 vs. control. (**B**) Effect of creatine treatment on *snf-5* expression. The adults were treated with 5 mM creatine for 24 h. Control, without 6-PPDQ exposure. ** *p* < 0.01 vs. control. (**C**) Effect of RNAi of *snf-5* on creatine content in 6-PPDQ-exposed nematodes. Exposure concentration of 6-PPDQ was 10 μg/L. ** *p* < 0.01.

Among genes encoding orthologs of creatine transporters, although 6-PPDQ exposure did not affect *snf-2*, *snf-3*, *snf-6*, *snf-7*, *snf-9*, *snf-11*, and *snf-12* expressions, *snf-5* expression was decreased by 0.1–10 μg/L 6-PPDQ (Figure 2A). The *snf-5* expression could be activated by creatine treatment (Figure 2B). Moreover, after 6-PPDQ exposure, creatine content was reduced by RNAi of *snf-5* (Figure 2C).

### 3.3. RNAi of argk-1 and snf-5 Aggravated 6-PPDQ Caused Immunosuppression

**Figure 3:**
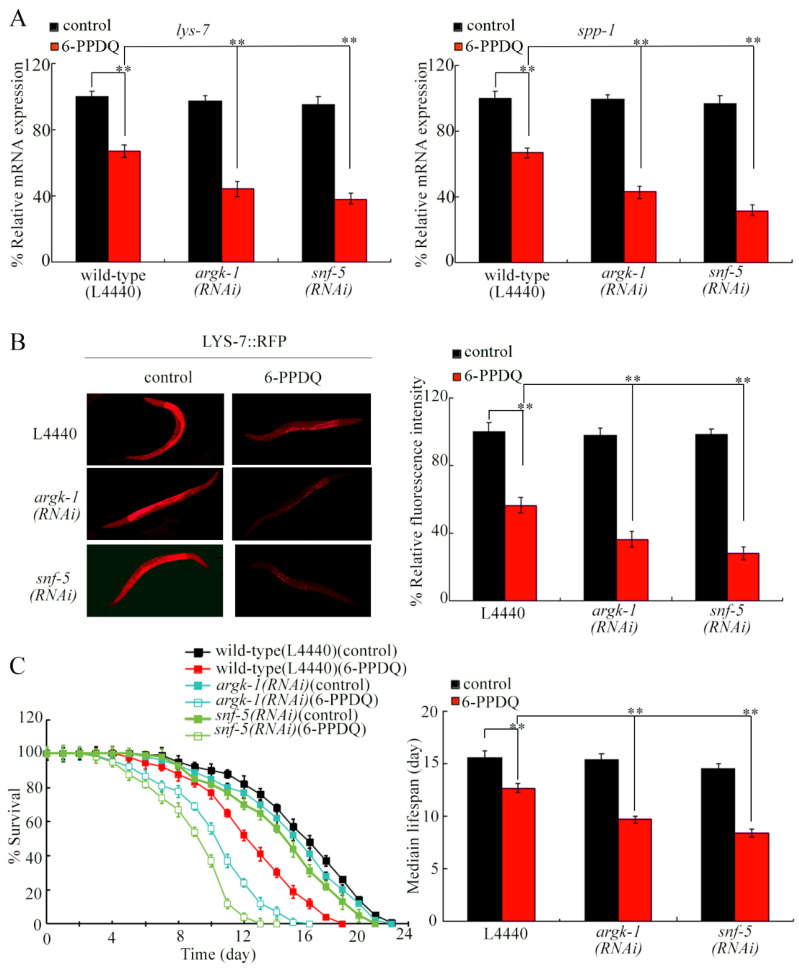
Effect of RNAi of *argk-1* and *snf-5* on 6-PPDQ-induced immunosuppression. (**A**) Effect of RNAi of *argk-1* and *snf-5* on *lys-7* and *spp-1* expressions in 6-PPDQ-exposed nematodes. (**B**) Effect of RNAi of *argk-1* and *snf-5* on LYS-7::RFP expression in 6-PPDQ-exposed nematodes. (**C**) Effect of RNAi of *argk-1* and *snf-5* on lifespan in 6-PPDQ-exposed nematodes. Lifespan curve of wild-type(6-PPDQ) was significantly (** *p* < 0.01) different from that of the wild-type(control). After 6-PPDQ exposure, lifespan curves of *argk-1(RNAi)* and *snf-5(RNAi)* were significantly (** *p* < 0.01) different from that of wild-type. Control, without 6-PPDQ exposure. Exposure concentration of 6-PPDQ was 10 μg/L. ** *p* < 0.01.

Expressions of *lys-7*, *spp-1*, and LYS-7::RFP were decreased by 6-PPDQ exposure (Figure 3A,B). The decrease in *lys-7*, *spp-1*, and LYS-7::RFP expressions by 6-PPDQ was aggravated by RNAi of *argk-1* and *snf-5* (Figure 3A,B). In addition, the lifespan reduction induced by 6-PPDQ was also strengthened by RNAi of *argk-1* and *snf-5* (Figure 3C).

### 3.4. Double RNAi of argk-1 and snf-5 Caused More Severe Immunosuppression in 6-PPDQ Exposed Nematodes

**Figure 4:**
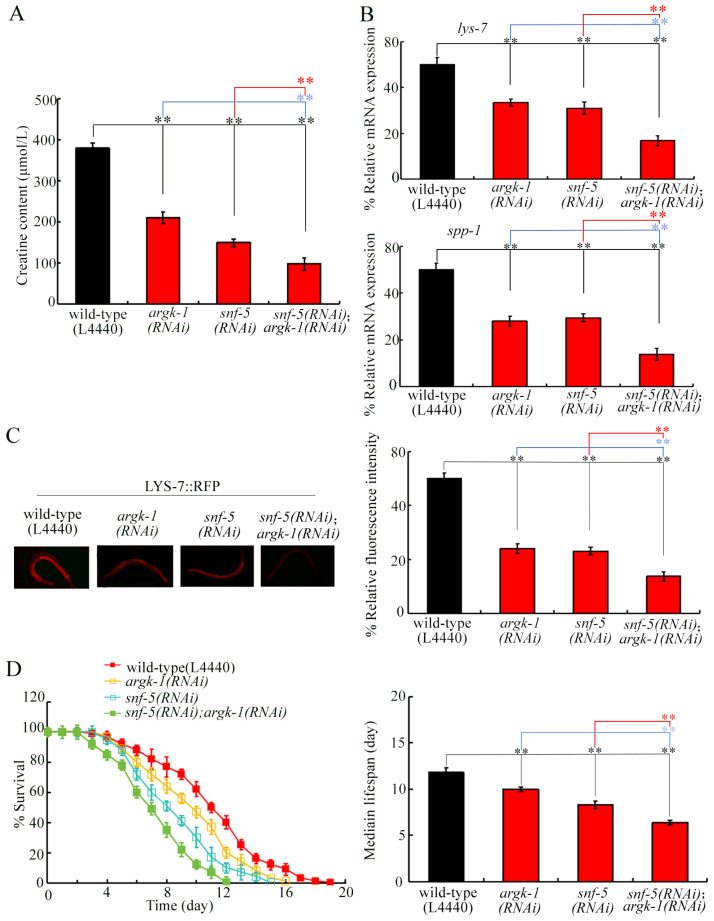
Genetic interaction between *argk-1* and *snf-5* in regulating 6-PPDQ-induced immunosuppression. (**A**) Genetic interaction between *argk-1* and *snf-5* in affecting creatine content in 6-PPDQ-exposed nematodes. (**B**) Genetic interaction between *argk-1* and *snf-5* on *lys-7* and *spp-1* expressions in 6-PPDQ-exposed nematodes. (**C**) Genetic interaction between *argk-1* and *snf-5* on LYS-7::RFP expression in 6-PPDQ-exposed nematodes. (**D**) Genetic interaction between *argk-1* and *snf-5* on lifespan in 6-PPDQ-exposed nematodes. After 6-PPDQ exposure, the lifespan curves of *argk-1(RNAi)* and *snf-5(RNAi)* were significantly (** *p* < 0.01) different from that of wild-type, and the lifespan curve of *snf-5(RNAi);argk-1(RNAi)* was significantly (** *p* < 0.01) different from those of *argk-1(RNAi)* and *snf-5(RNAi)*. Exposure concentration of 6-PPDQ was 10 μg/L. ** *p* < 0.01.

In 6-PPDQ-exposed nematodes, double RNAi of *argk-1* and *snf-5* caused a more severe reduction in creatine content compared to single RNAi of *argk-1* or *snf-5* (Figure 4A). Meanwhile, a more severe decrease in expressions of *lys-7*, *spp-1*, and LYS-7::RFP was observed in 6-PPDQ-exposed *snf-5(RNAi);argk-1(RNAi)* compared to those in 6-PPDQ-exposed *snf-5(RNA)* or *argk-1(RNAi)* (Figure 4B,C). In addition, more severe lifespan reduction was detected in 6-PPDQ-exposed *snf-5(RNAi);argk-1(RNAi)* compared to those in 6-PPDQ-exposed *snf-5(RNA)* or *argk-1(RNAi)* (Figure 4D).

### 3.5. RNAi of argk-1 Decreased aak-2 Expression in 6-PPDQ Exposed Nematodes

**Figure 5:**
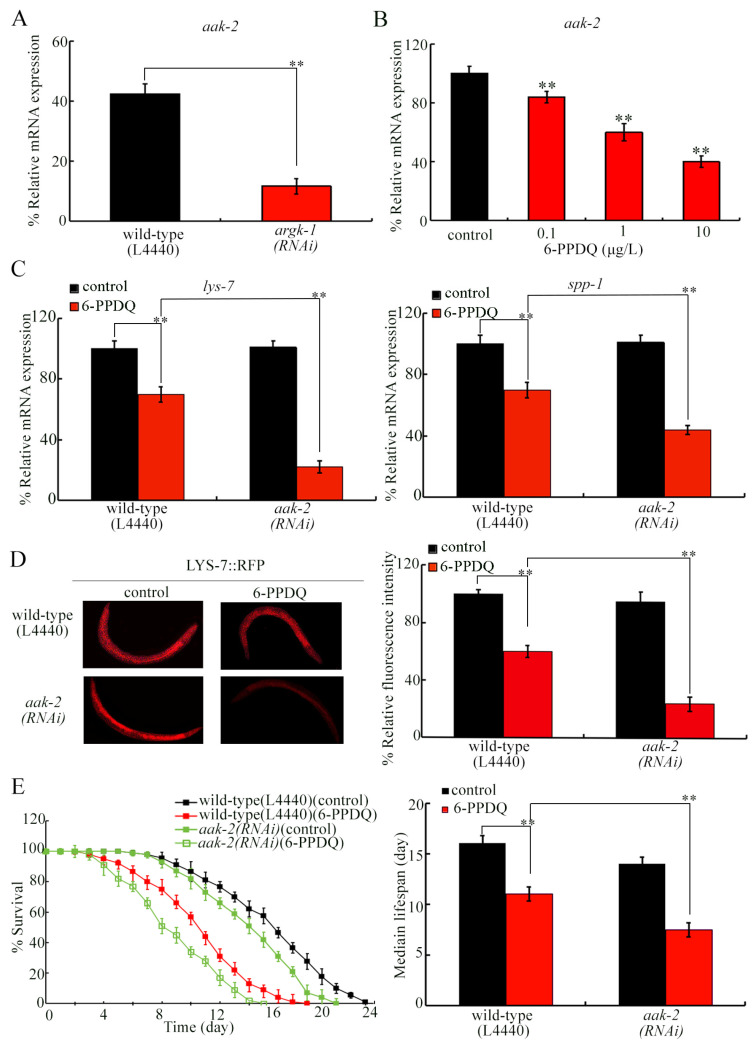
Effect of RNAi of *argk-1* on *aak-2* expression after 6-PPDQ exposure. (**A**) Effect of RNAi of *argk-1* on *aak-2* expression in 6-PPDQ-exposed nematodes. Exposure concentration of 6-PPDQ was 10 μg/L. ** *p* < 0.01. (**B**) Effect of 6-PPDQ exposure on *aak-2* expression. Control, without 6-PPDQ exposure. ** *p* < 0.01 vs. control. (**C**) Effect of RNAi of *aak-2* on *lys-7* and *spp-1* expressions in 6-PPDQ-exposed nematodes. Control, without 6-PPDQ exposure. Exposure concentration of 6-PPDQ was 10 μg/L. ** *p* < 0.01. (**D**) Effect of RNAi of *aak-2* on LYS-7::RFP expression in 6-PPDQ-exposed nematodes. Control, without 6-PPDQ exposure. Exposure concentration of 6-PPDQ was 10 μg/L. ** *p* < 0.01. (**E**) Effect of RNAi of *aak-2* on lifespan in 6-PPDQ-exposed nematodes. Lifespan curve of wild-type(6-PPDQ) was significantly (** *p* < 0.01) different from that of wild-type(control). After 6-PPDQ exposure, the lifespan curve of *aak-2(RNAi)* was significantly (** *p* < 0.01) different from that of wild-type. Control, without 6-PPDQ exposure. Exposure concentration of 6-PPDQ was 10 μg/L. ** *p* < 0.01.

Considering the important link between ARGK-1 and AAK-2 [32], we next examined the effect of *argk-1* RNAi on *aak-2* expression in 6-PPDQ-exposed nematodes. The *aak-2* expression was decreased by RNAi of *argk-1* and *snf-5* in 6-PPDQ-exposed nematodes (Figure 5A and Figure S2). The *aak-2* expression was reduced by 0.1–10 μg/L 6-PPDQ (Figure 5B). Moreover, 6-PPDQ caused a decrease in expressions of *lys-7*, *spp-1*, and LYS-7::RFP, which was aggravated by *aak-2* RNAi (Figure 5C,D). Similarly, 6-PPDQ-induced lifespan reduction was strengthened by *aak-2* RNAi (Figure 5E).

### 3.6. RNAi of argk-1, snf-5, and aak-2 Affected PMK-1 and DAF-16 Expressions in 6-PPDQ Exposed Nematodes

**Figure 6:**
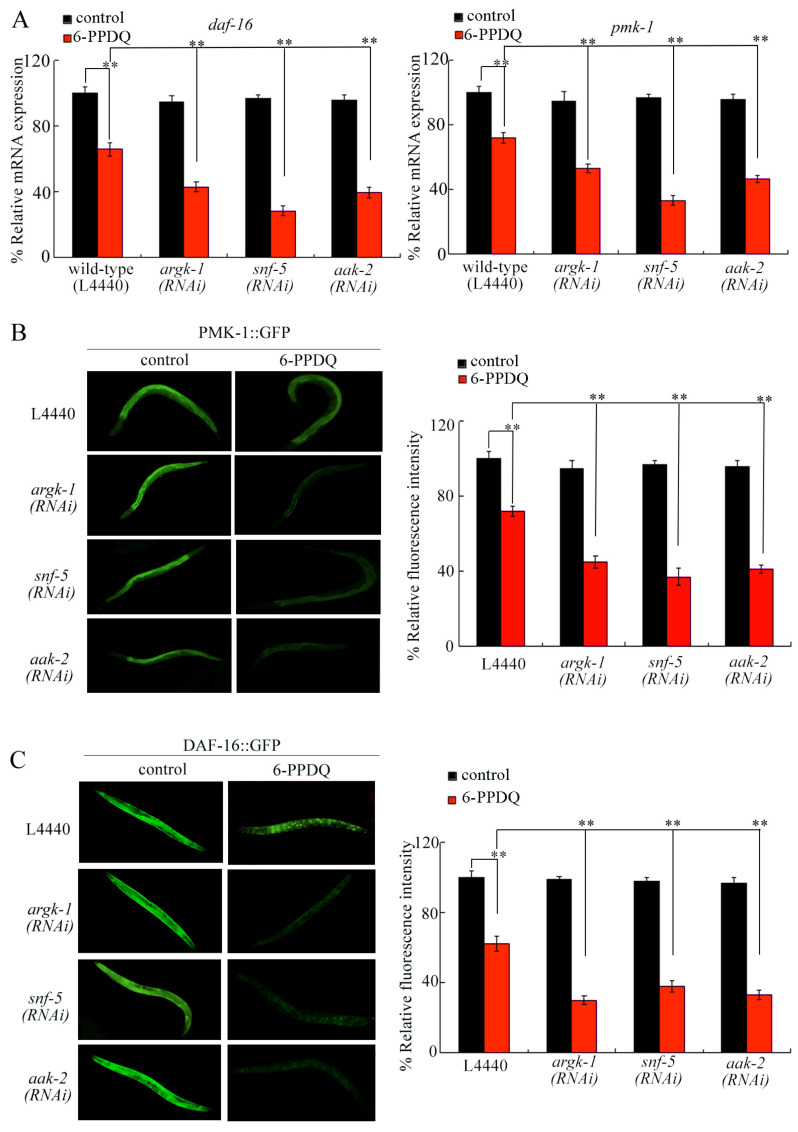
Effect of RNAi of *argk-1*, *snf-5*, and *aak-2* on expressions of DAF-16 and PMK-1 in 6-PPDQ-exposed nematodes. (**A**) Effect of RNAi of *argk-1*, *snf-5*, and *aak-2* on expressions of *pmk-1* and *daf-16* in 6-PPDQ-exposed nematodes. (**B**) Effect of RNAi of *argk-1*, *snf-5*, and *aak-2* on PMK-1::GFP expression in 6-PPDQ-exposed nematodes. (**C**) Effect of RNAi of *argk-1*, *snf-5*, and *aak-2* on DAF-16::GFP expression in 6-PPDQ-exposed nematodes. Control, without 6-PPDQ exposure. Exposure concentration of 6-PPDQ was 10 μg/L. ** *p* < 0.01.

Recently, PMK-1 and DAF-16 were identified as important molecular signals regulating 6-PPDQ caused immunosuppression [24]. Expressions of *pmk-1*, *daf-16*, PMK-1::GFP, and DAF-16::GFP were decreased by 6-PPDQ exposure (Figure 6A–C). 6-PPDQ caused decrease in *pmk-1*, and *daf-16* expressions were aggravated by RNAi of *argk-1*, *snf-5*, and *aak-2* (Figure 6A). Similarly, a 6-PPDQ-induced decrease in PMK-1::GFP and DAF-16::GFP expressions was strengthened by RNAi of *argk-1*, *snf-5*, and *aak-2* (Figure 6B,C). The 6-PPDQ-induced nuclear localization of DAF-16::GFP was increased by RNAi of *argk-1*, *snf-5*, and *aak-2* (Figure S3A).

### 3.7. Creatine Treatment Could Inhibit 6-PPDQ-Induced Immunosuppression

**Figure 7:**
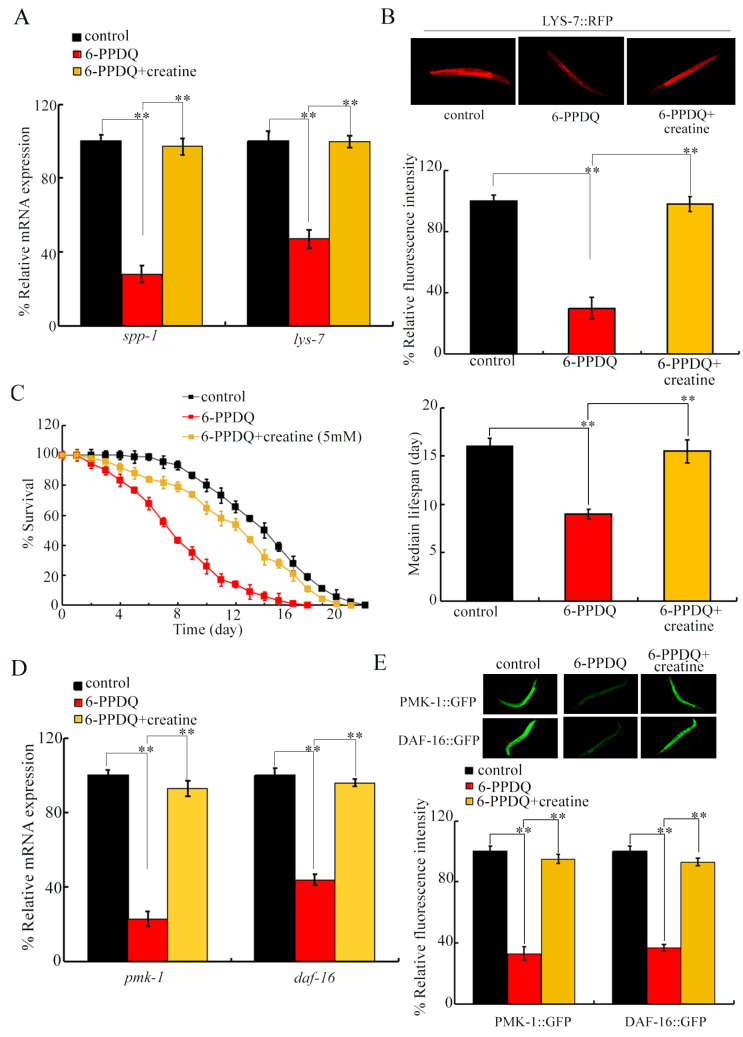
Pharmacological effect of creatine treatment on 6-PPDQ-induced immunosuppression. (**A**) Effect of creatine treatment on *lys-7* and *spp-1* expressions in 6-PPDQ-exposed nematodes. (**B**) Effect of creatine treatment on LYS-7::RFP expression in 6-PPDQ-exposed nematodes. (**C**) Effect of creatine treatment on lifespan in 6-PPDQ-exposed nematodes. Lifespan curve of 6-PPDQ was significantly (** *p* < 0.01) different from that of control, and the lifespan curve of 6-PPDQ + creatine was significantly (** *p* < 0.01) different from that of 6-PPDQ. (**D**) Effect of creatine treatment on *pmk-1* and *daf-16* expressions in 6-PPDQ-exposed nematodes. (**E**) Effect of creatine treatment on PMK-1::GFP and DAF-16::GFP expressions in 6-PPDQ-exposed nematodes. Following 6-PPDQ exposure (10 μg/L), nematodes were transferred into and treated with 5 mM creatine for 24 h. Control, without 6-PPDQ exposure and creatine treatment. ** *p* < 0.01.

To further confirm the role of creatine in modulating 6-PPDQ-induced immunosuppression, we further treated the 6-PPDQ-exposed nematodes with creatine. 6-PPDQ caused decrease in expressions of *spp-1* and *lys-7*, and LYS-7::RFP was suppressed by creatine treatment (Figure 7A,B). Similarly, 6-PPDQ-induced lifespan reduction was suppressed by creatine treatment (Figure 7C). Moreover, creatine treatment inhibited the decreasing tendency of *pmk-1*, *daf-16*, PMK-1::GFP, and DAF-16::GFP expressions induced by 6-PPDQ (Figure 7D,E). The 6-PPDQ-induced increase in nuclear localization of DAF-16::GFP was also inhibited by creatine treatment (Figure S3B).

## 4. Discussion

Creatine is important for maintaining cellular energy buffering, and it participates in rapid energy supply and energy redistribution through high-energy phosphate transfer [29]. In nematodes, we observed that exposure to 6-PPDQ at ERCs disrupted creatine synthesis, reflected by a reduction in creatine content (Figure 1B). ARGK-1 is a creatine kinase [32]. Inhibition of *argk-1* expression was found to act as a metabolic basis for the observed reduction in creatine content by 6-PPDQ exposure (Figure 1C). RNAi of *argk-1* decreased creatine content (Figure 1D), which confirms the function of *argk-1* in governing creatine synthesis. Considering that decreased creatine means reduced energy buffer capacity, 6-PPDQ may lower energy buffer capacity and impair the rapid regeneration of ATP. Recently, it was observed that there was no detectable metabolic conversion of creatine to creatinine in *C. elegans* [45]. Therefore, the possible metabolic processes of creatine in nematodes need to be further examined.

In mammals, SLC6A8 is the major creatine transporter, and its function is closely related to cellular creatine uptake and energy metabolism regulation by providing the sole gateway for creatine to enter cells [33]. The *C. elegans* SNF is homologous to the SLC6 family [34]. Among examined SNF family genes, *snf-5* was shown to be sensitive to 6-PPDQ exposure, and its expression was markedly decreased (Figure 2A). Meanwhile, exogenous creatine treatment significantly increased *snf-5* expression (Figure 2B), suggesting that *snf-5* may be associated with changes in creatine levels. Previous studies have annotated *snf-5* as a gene related to SLC6A8 homolog [35,36]. Further functional analysis showed that *snf-5* RNAi significantly decreased creatine content (Figure 2C). These results suggest that inhibition of SNF-5 also contributes to reduced creatine by 6-PPDQ exposure. Meanwhile, our data suggested that the expression of other transporter genes was not sensitive enough in response to 6-PPDQ at the examined concentrations.

We then analyzed whether *argk-1* and *snf-5* were involved in regulating immunosuppression induced by 6-PPDQ. *C. elegans* has conserved innate immune and stress defense networks, and antimicrobial peptide expression and lifespan changes have been widely used to evaluate immune damage and toxic effects induced by environmental pathogens or pollutants [15]. RNAi of *argk-1* and *snf-5* aggravated the decrease in expression of antimicrobial genes of *lys-7* and *spp-1* induced by 6-PPDQ (Figure 3A,B). In addition, RNAi of *argk-1* and *snf-5* further shortened the lifespan of nematodes exposed to 6-PPDQ (Figure 3C). That is, inhibition of ARGK-1 and SNF-5 mediated 6-PPDQ caused immunosuppression. Moreover, 6-PPDQ caused immunosuppression and lifespan reduction could be inhibited by creatine treatment (Figure 7A–C). These observations demonstrate the important role of creatine and related ARGK-1 and SNF-5 signals in controlling immunity in 6-PPDQ-exposed nematodes. Creatine uptake can also promote CD8^+^ T cell antitumor immunity [30], and creatine transport and creatine kinase activity are required for CD8^+^ T cell immunity [46]. In the current study, lifespan was used as an indirect indicator to reflect the immunosuppression induced by 6-PPDQ. In addition to this, RNAi of *lys-7* and *spp-1* also aggravated 6-PPDQ caused generation of reactive oxygen species (ROS) production and enhancement in intestinal permeability [23]. That is, ROS production and intestinal permeability can also be employed as indirect indicators for immunosuppression induction.

To further clarify the interaction between *snf-5* and *argk-1*, we performed double RNAi analysis. Under 6-PPDQ exposure condition, double RNAi of *argk-1* and *snf-5* caused a stronger decrease in creatine content than single RNAi treatment (Figure 4A). In terms of immune response, double RNAi also further aggravated 6-PPDQ caused immunosuppression (Figure 4B,C) and lifespan reduction (Figure 4D). These results imply that *snf-5* and *argk-1* may function synergistically to regulate 6-PPDQ toxicity on immunity, which was associated with their function in affecting creatine content. More importantly, this implies an important toxicity-amplifying mechanism.

ARGK-1 can regulate longevity by activating energy sensor AAK-2/AMPK [32]. 6-PPDQ at ERCs decreased *aak-2* expression (Figure 5B). Meanwhile, under 6-PPDQ exposure condition, *argk-1* and *snf-5* RNAi decreased *aak-2* expression (Figure 5A and Figure S2). AAK-2 acted as an energy-sensing signal to participate in the control of innate immune response in *C. elegans* [47]. Energy sensor AAK-2 may provide an important linker between ARGK-1 and immunosuppression in 6-PPDQ-exposed nematodes. The 6-PPDQ caused immunosuppression and lifespan reduction, which were further strengthened by RNAi of *aak-2* (Figure 5C–E). Therefore, energy sensor AAK-2 may participate in maintaining immune defense status in 6-PPDQ-exposed nematodes.

Recently, we identified PMK-1 and DAF-16 as regulators for 6-PPDQ caused immunosuppression, and 6-PPDQ induced immunosuppression by inhibiting PMK-1 and DAF-16 [24]. PMK-1 and DAF-16 play a central role in regulating *C. elegans* immunity [48,49]. Moreover, a decrease in *pmk-1*, *daf-16*, PMK-1::GFP, and DAF-16::GFP expressions by 6-PPDQ exposure were aggravated by RNAi of *argk-1*, *snf-5*, and *aak-2* (Figure 6). More importantly, the 6-PPDQ-induced decrease in *pmk-1*, *daf-16*, PMK-1::GFP, and DAF-16::GFP expressions could be inhibited by creatine treatment (Figure 7D,E), which further suggests that creatine metabolic disturbance induced by 6-PPDQ may be associated with immunosuppression by inhibiting PMK-1 and DAF-16 signals. In 6-PPDQ-exposed nematodes, accompanied by a decrease in DAF-16::GFP expression, we observed an increase in nuclear localization of DAF-16::GFP (Figure S3). This represents a compensatory stress response, where the remaining DAF-16 proteins are more actively translocated into the nucleus to mount a transcriptional defense against 6-PPDQ-induced toxicity. Due to a lack of corresponding *C. elegans* antibodies, we did not perform further assays on PMK-1 phosphorylation after 6-PPDQ exposure.

## 5. Conclusions

Altogether, we observed a reduction in creatine content by 6-PPDQ at ERCs in nematodes. This reduction in creatine content by 6-PPDQ was due to inhibition in the expression of both creatine kinase gene *argk-1* and transporter gene *snf-5*. Moreover, both inhibition in ARGK-1 and inhibition in SNF-5 were associated with 6-PPDQ, which caused immunosuppression by modulating PMK-1 and DAF-16 signals. Pharmacological treatment with creatine further indicated the role of creatine in modulating 6-PPDQ, which caused immunosuppression. Therefore, our observations highlight the important link between disruption in creatine synthesis and transporter and 6-PPDQ caused immunosuppression in nematodes. These findings in *C. elegans* suggest that 6-PPDQ-induced immunosuppression is associated with disrupted creatine synthesis and transporter, which may offer the conserved mechanistic biomarkers and potential therapeutic targets for assessing similar immunotoxic risks in mammals. Nevertheless, the confirmation of the role of creatine synthesis and transporter is suggested to be further performed in mammals.

## Data Availability

The original contributions presented in this study are included in the article/Supplementary Materials. Further inquiries can be directed to the corresponding author.

## Supplementary Materials

The following supporting information can be downloaded at https://www.mdpi.com/article/10.3390/toxics14070601/s1. Figure S1: RNAi efficiency of snf-5, argk-1, and aak-2; Figure S2: Effect of RNAi of snf-5 on aak-2 expression in 6-PPDQ exposed nematodes; Figure S3: Change of DAF-16::GFP nuclear localization; Table S1: Information for *C. elegans* strains; Table S2: Primer information for qRT-PCR.
