# Supplementary material for: Contribution of Disruption in Creatine Synthesis and Transporter to 6-PPD Quinone Induced Immunosuppression in Caenorhabditis elegans
Source: Toxics. 2026 Jul 9;14(7):601. doi: 10.3390/toxics14070601 (PMC13416720; doi:10.3390/toxics14070601)
Supplement: Supplementary file 1 [file toxics-14-00601-s001.zip › toxics-4403739-supplementary.pdf]

Supporting Information:

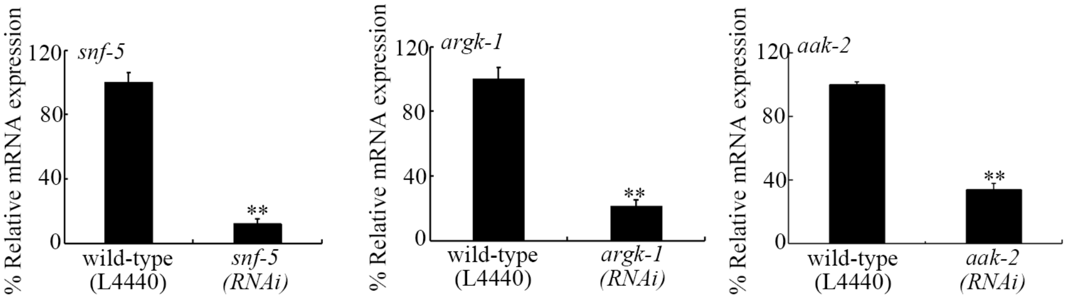

Figure S1 RNAi efficiency of *snf-5*, *argk-1*, and *aak-2*. $^{**}P < 0.01$ *vs* wild-type(L4440).

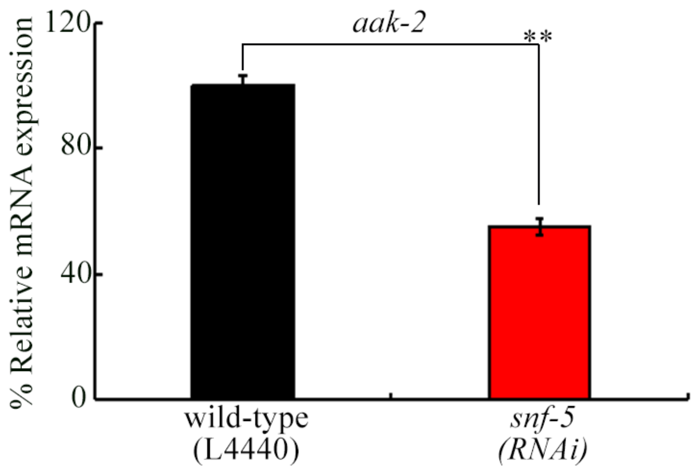

Figure S2 Effect of RNAi of *snf-5* on *aak-2* expression in 6-PPDQ exposed nematodes. Exposure concentration of 6-PPDQ was 10 μg/L. $^{**}P < 0.01$.

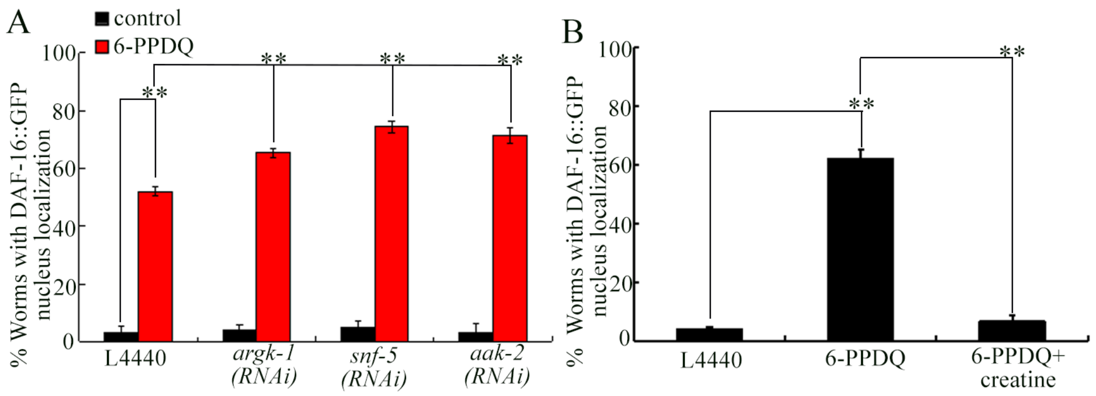

Figure S3 Change of DAF-16::GFP nuclear localization. (A) Effect of *argk-1*, *snf-5*, and *aak-2* on DAF-16::GFP nuclear localization in 6-PPDQ exposed nematodes. Control, without 6-PPDQ exposure. Exposure concentration of 6-PPDQ was 10 μg/L. $^{**}P < 0.01$. (B) Effect of creatine treatment on DAF-16::GFP nuclear localization in 6-PPDQ exposed nematodes. Following 6-PPDQ exposure (10 μg/L), nematodes were transferred into and treated with 5 mM creatine for 24 h. Control, without 6-PPDQ exposure and creatine treatment. $^{**}P < 0.01$.

Table S1 Information for *C. elegans* strains

Strains	Genotype	Description
N2		Wild-type
TJ356	*zIs356*[*daf-16p::daf-16a/b::GFP+ rol-6(su1006)*]	Transgenic strain expressing DAF-16::GFP
PRJ112	*mutEx70* [*pmk-1::GFP* + *rol-6(su1006)*]	Transgenic strain expressing PMK-1::GFP
CF2018	*muEx304 [lys-7p::RFP(NLS) + rol-6(su1006)]*	Transgenic strain expressing LYS-7::RFP

Table S2 Primer information for qRT-PCR

Gene	Forward primer (5'-3')	Reverse primer (5'-3')
snf-2	TGTGGAGTTAGCTTGCCTGG	GCACTGGCATTTCAGCGATT
snf-3	TTACACATTCCGGCTCCCAC	GGGATGACAACCATGGAGCA
snf-5	CAATGAAACGCCAAGCACCA	GCCACGAATACACCCATCCA
snf-6	TCTGGGCTCTTCTGGATGGA	CAATTTCGACTTCTGGCGGC
snf-7	CCGTGATTCCTCCAACTGCT	ACTTGGCTGCATACTGTCCC
snf-9	GAAAGTGGCTGGTTGCAGTG	GGTAGAAGACAAACCGCCCA
snf-11	AAATCCAGGTGGCATCCGTT	CGCTTGAGCTACTTCGGCTA
snf-12	GGATGGCTCGTAAAACACGC	ACCTGCCATTCTTCTGCTCC
lys-7	TCGGCATCAGTCAAGGTTCC	TGGCGAAGTGACCTGAATCC
spp-1	CGATTCCTTATCAGTGCCGC	ACGGCAACAGCATAGTCCAG
pmk-1	TCCGACTCCACGAGAAGGAT	CACGATATGTACGACGGGCA
daf-16	ACATTGCTCGAAGTGCCGAA	CATTGCTGTCGACCCGTTTG
argk-1	GACGAGGAACTCAAGGGGAC	CGAGCGCTGAGTTTTGGAAG
aak-2	TCTTCCGCCATCCGCATATC	CCTCTTCATCGGGTCTACGC
tba-1	TCAACACTGCCATCGCCGCC	TCCAAGCGAGACCAGGCTTCAG
